# Characterization of Free Exopolysaccharides Secreted by *Mycoplasma mycoides* Subsp. *mycoides*

**DOI:** 10.1371/journal.pone.0068373

**Published:** 2013-07-15

**Authors:** Clothilde Bertin, Corinne Pau-Roblot, Josiane Courtois, Lucía Manso-Silván, François Thiaucourt, Florence Tardy, Dominique Le Grand, François Poumarat, Patrice Gaurivaud

**Affiliations:** 1 Agence Nationale de Sécurité Sanitaire, Laboratoire de Lyon, UMR Mycoplasmoses des Ruminants, Lyon, France; 2 Université de Lyon, VetAgro Sup, UMR Mycoplasmoses des Ruminants, Marcy-L’étoile, France; 3 Unité Biologie des Plantes et Innovation, EA 3900, Université de Picardie Jules Verne, Amiens, France; 4 Centre International de Recherche en Agronomie pour le Développement, UMR CMAEE, Montpellier, France; 5 Institut National de Recherche Agronomique, UMR1309 CMAEE, Montpellier, France; Miami University, United States of America

## Abstract

Contagious bovine pleuropneumonia is a severe respiratory disease of cattle that is caused by a bacterium of the Mycoplasma genus, namely *Mycoplasma mycoides* subsp. *mycoides* (*Mmm*). In the absence of classical virulence determinants, the pathogenicity of *Mmm* is thought to rely on intrinsic metabolic functions and specific components of the outer cell surface. One of these latter, the capsular polysaccharide galactan has been notably demonstrated to play a role in *Mmm* persistence and dissemination. The free exopolysaccharides (EPS), also produced by *Mmm* and shown to circulate in the blood stream of infected cattle, have received little attention so far. Indeed, their characterization has been hindered by the presence of polysaccharide contaminants in the complex mycoplasma culture medium. In this study, we developed a method to produce large quantities of EPS by transfer of mycoplasma cells from their complex broth to a chemically defined medium and subsequent purification. NMR analyses revealed that the purified, free EPS had an identical β(1−>6)-galactofuranosyl structure to that of capsular galactan. We then analyzed intraclonal *Mmm* variants that produce opaque/translucent colonies on agar. First, we demonstrated that colony opacity was related to the production of a capsule, as observed by electron microscopy. We then compared the EPS extracts and showed that the non-capsulated, translucent colony variants produced higher amounts of free EPS than the capsulated, opaque colony variants. This phenotypic variation was associated with an antigenic variation of a specific glucose phosphotransferase permease. Finally, we conducted *in silico* analyses of candidate polysaccharide biosynthetic pathways in order to decipher the potential link between glucose phosphotransferase permease activity and attachment/release of galactan. The co-existence of variants producing alternative forms of galactan (capsular versus free extracellular galactan) and associated with an antigenic switch constitutes a finely tuned mechanism that may be involved in virulence.

## Introduction

Bacteria of the Mycoplasma genus have evolved from their Gram-positive ancestors by massive reduction of their genome size. They are considered the smallest and simplest self-replicating organisms, with many missing biosynthetic pathways and the notable absence of a cell wall [1]. Despite this apparent simplicity, mycoplasmas are able to develop metabolic responses and adaptations similar to those of more complex bacteria, due notably to their higher proportion of multifunctional enzymes [2]. Several mycoplasma species are known to cause important diseases in humans and animals. Members of the so-called *Mycoplasma mycoides* cluster are particularly deleterious for ruminants and are responsible for severe economic losses worldwide. *Mycoplasma (M.) mycoides subsp. mycoides* (*Mmm*) is the causal agent of contagious bovine pleuropneumonia (CBPP), a highly contagious respiratory disease notifiable to the World Organization for Animal Health (Office International des Epizooties, OIE). CBPP was formerly one of the major cattle diseases worldwide and has now been eradicated from many countries, although it persists in Africa. Under natural conditions, CBPP affects only the *Bos* genus, producing pathognomonic clinical lesions confined to the thoracic cavity [3]. Contrary to other mycoplasmas involved in cattle respiratory diseases, such as *M. bovis* [4], *Mmm* is not considered an agent of co-infection and is often isolated on its own. This clinical picture contrasts with that observed in infections *by M. mycoides* subsp. *capri,* its closest phylogenetic neighbor and one of the agents of contagious agalactia, a small-ruminant syndrome affecting diverse organs (udder, joints, eyes, lungs).

None of the usual virulence determinants described for other bacteria, such as toxins, invasins or cytolysins, has been identified in the currently sequenced *Mmm* genomes [5], [6]. However, several mechanisms have been proposed to account for *Mmm* pathogenicity such as adherence to the host tissues, immune evasion, persistence, dissemination, inflammation and cytotoxicity [7]. The polysaccharides have been often implicated in the pathogenesis of bacteria colonizing the lung [8] and have attracted attention for decades as a potential *Mmm* virulence factor. *Mmm* has been shown to produce two types of polysaccharides: a capsular one, namely galactan, identified [9] and chemically characterized [10] by Buttery and Plackett in the 1960s, and an extracellular one, hereafter named exopolysaccharide (EPS) as defined by Branda and collaborators [11]. The purification of free EPS has been jeopardized by polysaccharide contaminants in the complex culture medium used to grow mycoplasmas. EPS is immunologically related to the capsular galactan and has been found in large amounts as a free *Mmm* product unassociated with cells, both in culture supernatants and in the blood of infected cattle [12]. *Mmm* strains able to produce large amounts of capsular polysaccharide proved less sensitive to growth inhibition by bovine antisera [13] and displayed longer bacteremia than strains with little capsular polysaccharide in a mouse infection model [14]. This clearly pointed towards a role of capsular polysaccharides in *Mmm* persistence and dissemination. Antigenic cross-reactivity between *Mmm* capsular galactan and the pneumogalactan isolated from bovine lung was proposed to be at the origin of autoimmune reactions [15] and thus to participate in the inflammation process. In addition, intravenous injection of free, extracellular EPS induced specific effects on the vascular system of the lung and respiratory system of cattle [16] and promoted persistent bacteremia in calves, when it preceded inoculation of an *Mmm* strain [17], [18].

The present study was conducted to elucidate the composition and structure of the EPS produced by *Mmm* and to compare it with the capsular galactan. We developed a purification method consisting of transferring mycoplasma cells from their complex growth medium to a chemically defined medium to minimize polysaccharide contamination. Two strains were used: (i) the type strain PG1^T^, for which the complete genome sequence is available [5], and (ii) the field strain Afadé, highly virulent and already used for biofilm formation [19] and virulence studies [20], as it causes CBPP under natural and experimental conditions [21]. EPS purified from the supernatant of *Mmm* strain Afadé proved to have the same composition and structure as capsular galactan and was also recognized by CBPP-convalescent bovine serum. Furthermore, the production of capsular versus free galactan was associated with variations in colony opacity related to an antigenic switch of a specific glucose permease. *In silico* analyses were conducted in order to decipher the metabolic pathways that might account for the production of either capsular galactan or free EPS by the corresponding opaque and translucent variants.

## Materials and Methods

### 1. Strains, Variant Selection and Growth Conditions

Two strains were used in this study: *Mmm* Afadé (From the CIRAD collection, isolated from cattle in Chad in 1968), used for EPS purification and for selection of opaque (OP) and translucent (TR) colony variants and PG1^T^ (type strain, NCTC 10114), used for electron microscopy and for *in silico* analysis of galactan biosynthetic pathways. Both strains were grown at 37°C under 5% CO_2_ in PPLO-based medium (DIFCO) supplemented as described previously [22]. The chemically defined CMRL-1066 medium (Invitrogen), supplemented with amoxicillin 2 g/L (GlaxoSmithKline), was used for exopolysaccharide production. OP and TR colony variants of strain Afadé were selected on PPLO agar medium and sub-cultured to check their homogeneity. They were also grown on 30 µg/ml Congo red plates to visualize any extracellular material. OP and TR colonies were analyzed by colony blotting as described previously [23]. In brief, the monoclonal antibody 3F3 [24] was used to detect TR colonies, while OP colonies were counterstained with Ponceau red. The proportion of each variant was estimated by colony counting after blot staining.

### 2. EPS Production and Purification

Fifty mL of PPLO-based medium were inoculated with 500 µL of mycoplasma culture in stationary phase and incubated for 2 days at 37°C in 5% CO_2._ Mycoplasma cell density was estimated by cell enumeration using the most probable number method [25]. Cells were harvested by centrifugation at 12000 g for 45 min at 20°C, washed once with 20 mL sterile PBS (10 mM sodium phosphate, 150 mM NaCl, pH 7.4) and finally suspended in 500 µL sterile PBS. This suspension was used to inoculate PPLO-based broth or CMRL-1066 medium with 10^8^–10^10^ mycoplasmas/mL. The cultures were further incubated for 72 h to 96 h. Aliquots were sampled at 24 h intervals to measure pH and cell density and to purify EPS.

EPS were purified as described by De Vuyst et al. [26], with a few modifications. Cells were removed by centrifugation (14000 g, 45 min, 4°C). A proteinase K (Promega) treatment (60 µg/mL for 4 hours at 37°C) was applied only as an extraction control from the PPLO-based medium in order to degrade serum proteins associated with EPS. Polypeptides were precipitated with 1/10 volume of cold 100% (w/v) trichloroacetic acid (Sigma-Aldrich) at 4°C for 2 hours followed by centrifugation (14000 g, 60 min, 4°C). Polysaccharides contained in the supernatant were precipitated with 6 to 10 volumes of cold acetone at −20°C for 48 h and collected after centrifugation at 14000 g, 60 min at 4°C. The acetone was carefully removed and the pellet was air-dried and dissolved in ultra-pure sterile water. For NMR and monomer composition analysis, extracts were dialyzed against regularly-renewed ultra-pure sterile water for 48 hours at room temperature in 3.5 kDa cut-off dialysis tubing (Spectrum Laboratories).

The effect of carbon sources on EPS production by strain Afadé was studied by adding to CMRL-1066 medium, which already contains 1 g/L glucose, either (i) 4 g/L glucose, mannitol, galactose, sorbitol or sucrose before seeding or (ii) 1 g/L glucose at 24, 48 and 72 hours after seeding. Carbohydrates were prepared as 200 g/L stock solutions and sterilized by filtration (0.22 µm filter units) before addition to CMRL-1066.

### 3. EPS Quantification and Characterization

The concentration of total sugars was estimated by phenol/sulfuric acid method [27] using glucose as standard. For each sample, the determination was done in triplicate and results were expressed as µg of glucose/mL of CMRL-1066 mean +/− standard deviation (SD). The potential presence of polypeptides in the EPS extract was assessed by silver staining of Any kD polyacrylamide gel (Bio-Rad) using the proteosilver kit from Sigma-Aldrich.

EPS was detected with a polysaccharide detection kit and immunodetected by dot blotting using a CBPP positive serum. In brief, 2µL of the EPS extract were spotted onto a nitrocellulose membrane that was either stained with the glycoprotein detection kit from Sigma-Aldrich or treated as previously described for dot immunobinding [28].

### 4. Electron Microscopy

Electron microscopy was performed as previously described [29] using five-day-old colonies of *Mmm* strain PG1^T^ grown on solid PPLO-based medium. Samples were examined under a Philips CM120 transmission electron microscope operating at an accelerating voltage of 75 kV.

### 5. Monosaccharide Composition Determination

The monosaccharide components were determined by high-performance anion exchange chromatography (HPAEC) on a CarboPak PA 1 with a pulsed amperometric detector (Dionex ICS 3000 system). After hydrolysis of EPS (1 mg) with 4 M CF_3_CO_2_H (100°C, 4 h), aliquots of the extract were passed through a 4×50 mm Propac PA1 pre-column (Dionex) before separation of anionic compounds on a 4×250 mm Propac PA1 column (Dionex) at 30°C. Gradient elution was performed with a multi-step gradient as follows: 0–25 min, 90% H_2_O and 10% NaOH 160 mM; 25–34 min, 100% NaOH 200 mM; 35–50 min 90% H_2_O and 10% NaOH 160 mM at a flow rate of 1 mL/min. Peak analysis was performed using Chromeleon software, version 7.0.

### 6. NMR Spectroscopy

Prior to NMR analysis, samples were exchanged twice with 99.9% D_2_O (Euriso-top), dried under vacuum, and dissolved in 99.96% D_2_O (<1 mg/0.5 mL). ^1^H NMR spectra were recorded, at 80°C, on a Bruker Avance 500 spectrometer equipped with a 5 mm BBI probe and Topspin 1.3 software. ^1^H NMR spectra were accumulated using a 30° pulse angle, a recycle time of 1 s and an acquisition time of 2 s for a spectral width of 3 000 Hz for 32 K data points with presaturation of the HOD signal using a presaturation sequence provided by Bruker. ^13^C NMR experiments were conducted on the same spectrometer operating at 125.48 MHz with 2 s as relaxation delay.

The 2D ^1^H/^1^H COSY, ^1^H/^1^H TOCSY, ^1^H/^1^H NOESY, ^1^H/^13^C HSQC and ^1^H/^13^C HMBC spectra were acquired with standard pulse sequences delivered by Bruker.

### 7. *In silico* Analysis of Candidate Galactan Biosynthetic Pathways

Genes potentially involved in EPS biosynthetic pathways were retrieved from the molligen database [30] and from the Carbohydrate-Active EnZymes database (CAZy) [31]. Potential transmembrane regions were predicted by TMHMM2 [32]. Putative functions and conserved domains of proteins encoded by genes were analyzed using the profile HMM method [33], [34] and PSI-BLAST [35].

## Results and Discussion

### *1. Mmm* Growth and EPS Secretion in Defined Medium versus Complex Mycoplasma Broth

**Figure 1:**
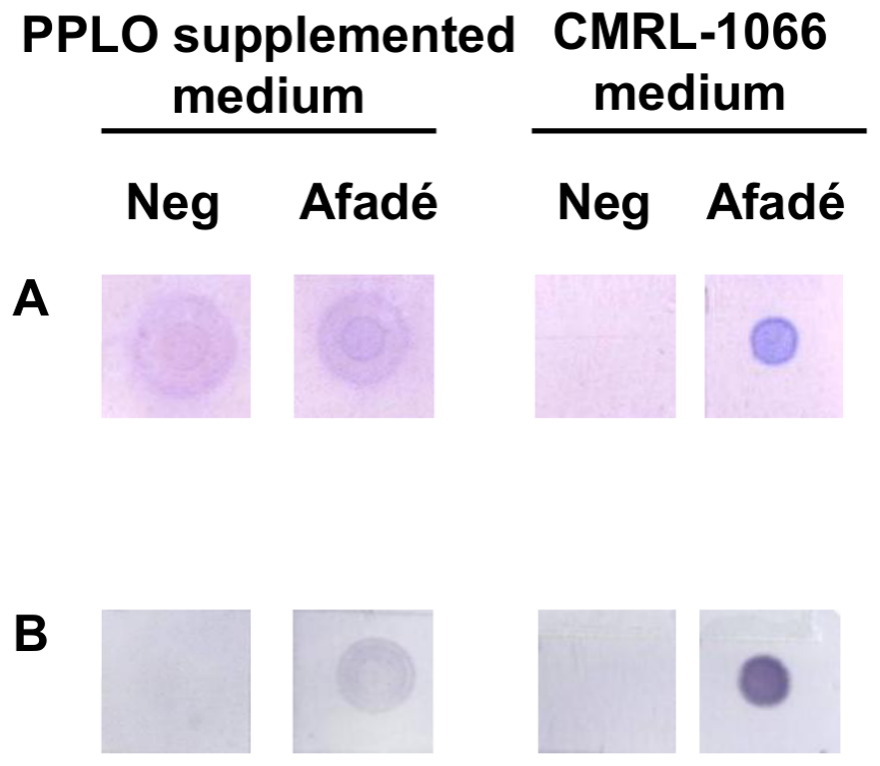
Exopolysaccharide (EPS) secretion by *Mmm* strain Afadé in supplemented PPLO broth and CMRL-1066 medium. EPS extracted either from *Mmm* culture (Afadé) or from unseeded medium used as negative control (Neg) were spotted onto a nylon membrane and either stained with a glycoprotein detection reagent (**A**) or immunodetected using a serum from a CBPP-positive bovine (**B**).

It had been previously shown that large amounts of polysaccharides not bound to the cells could be recovered from *Mmm* cultures [17], [36]. However, at the time, these polysaccharides could not be purified from the complex, undefined medium used to grow mycoplasmas. In this work we developed an experimental procedure that limits the potential contamination of newly synthesized polysaccharides by saccharides from the growth medium. Mycoplasma cells were first grown to stationary phase in a classic PPLO-based medium, then washed and transferred to a chemically defined, synthetic medium (CMRL-1066) for further incubation. CMRL-1066 is a constituent of the commonly used mycoplasma SP4 medium [37] and contains all the components needed to support cellular metabolism, but not growth. Following a 72 h-incubation in CMRL-1066, polysaccharide extracts were clearly detected in culture supernatants of *Mmm* strain Afadé, while no polysaccharides were detected in non-inoculated CMRL-1066 used as negative control (Figure 1A). These polysaccharides were not attached to the cells and were therefore considered as EPS [11]. In contrast, polysaccharide production by *Mmm* could not be assessed in PPLO-based medium because of detection of a strong background in the negative control corresponding to the saccharides contained in this growth medium (Figure 1A).

**Figure 2:**
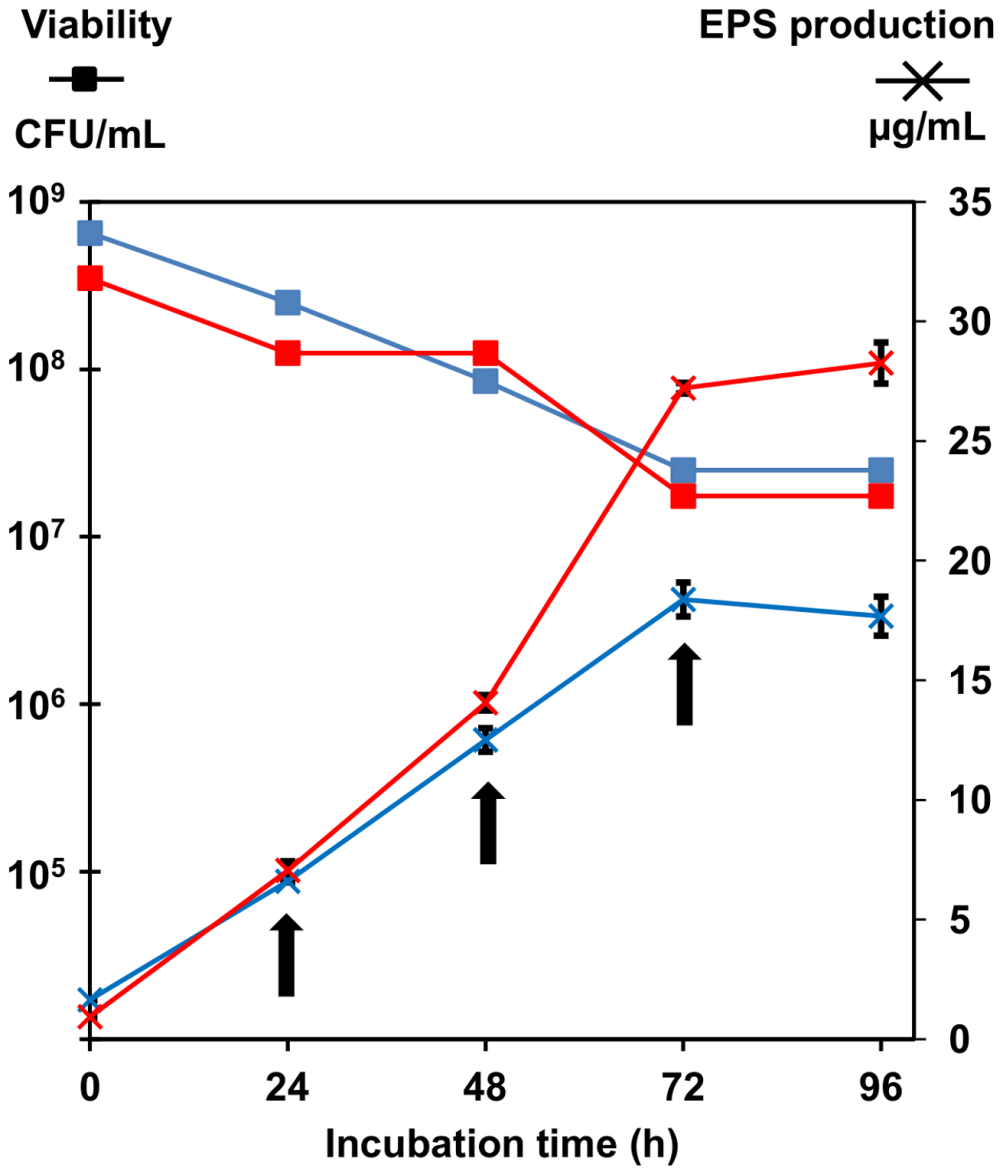
Kinetics of exopolysaccharides secretion and viability of ***Mmm*** **strain Afadé following transfer into CMRL-1066 medium with (red) or without (blue) glucose supplementation.** Glucose (1 g/L) was added at 24, 48 and 72 h (arrows).

EPS production in CMRL-1066 was shown to be reproducible, maximum after 72 h of incubation and related to the initial viable mycoplasma titer (Figure 2, Table 1). EPS detection proved to be difficult when CMRL-1066 was inoculated with less than 10^8^ mycoplasmas/mL. CMRL-1066 was not able to sustain mycoplasma growth and a decrease in the number of viable cells was observed during the incubation period (Figure 2, Table 1). This decrease in viability was not related to a high inoculum concentration, as it was also evidenced in several serial ten-fold dilutions of the initial culture (Table 1). The poor cholesterol concentration (0.2 mg/L) and the absence of other fatty acids in CMRL-1066 might result not only in growth inhibition but also in cell lysis, as previously suggested [38]. In turn, it can be hypothesized that this cellular decay may artificially contribute to EPS release into the culture supernatant [39]. However, no correlation was found between loss of viability and EPS production after 72 h of incubation (data not shown), suggesting that the contribution of cell lysis to EPS release was minor.

**Table 1:** Exopolysaccharide secretion and viability of *Mmm* strain Afadé in CMRL-1066 medium at the time of inoculation (T0) and after 72 h of incubation (T72).

		CFU/mL		
Strain/variant	Sugar added (4 g/L)	T0	T72	EPS µg/mL T72
Afadé	None	4.8×10^10^	4.0×10^8^	48.8
	None	a(3.6+/−1.0)x10^9^	a(2.1+/−1.0)x10^8^	a25.2+/−3.0
	None	a(4.9+/−0.8)x10^8^	a(8.9+/−7.0)x10^6^	a8.2+/−4.4
	None	a(4.0+/−0.5)x10^7^	a(1.4+/−0.2)x10^5^	a1.0+/−0.5
	None	4.8×10^6^	4.0×10^4^	0.5
	None	4.8×10^5^	3.0×10^1^	0.6
	None	6.5×10^9^	1.7×10^6^	24.0
	Glucose	6.5×10^9^	2.5×10^6^	31.8
	Sorbitol	6.5×10^9^	6.5×10^5^	27.9
	Mannitol	6.5×10^9^	1.7×10^7^	37.0
	Galactose	6.5×10^9^	8.5×10^6^	28.5
	Sucrose	6.5×10^9^	4.0×10^7^	37.5
TR variant	None	a(3.4+/−0.5)×10^8^	a(6.1+/−5.3)×10^6^	a15.4+/−1.1
OP variant	None	a(6.0+/−2.0)×10^8^	a(2.7+/−0.6)×10^8^	a2.9+/−1.0
	None	6.5×10^8^	1.2×10^8^	0.7
	Glucose	6.5×10^8^	8.5×10^7^	2.3
	Sorbitol	6.5×10^8^	1.2×10^8^	0.5
	Mannitol	6.5×10^8^	4.0×10^5^	0.9
	Galactose	6.5×10^8^	2.0×10^7^	0.7
	Sucrose	6.5×10^8^	1.2×10^8^	1.9

a Data correspond to mean +/− standard deviation of several (> = 3) experiments.

In 1972 Sutherland suggested that bacterial growth inhibition may result in a rise in EPS production due to the increased availability of EPS precursors, not used for glycolysis [40]. This was explored here by analysis of CMRL-1066 supplementation with different sugars during incubation. Regular addition of glucose (1 g/L) had no effect on mycoplasma viability but resulted in a 50% increase in EPS production (Figure 2). A similar increase of EPS production was measured when 4 g/L of glucose were added to CMRL-1066 at the beginning of incubation (Table 1). This indicated that during incubation in minimal medium unable to sustain growth, glucose was not catabolized by mycoplasmas but was used to produce EPS. Supplementation of CMRL-1066 medium, inoculated with 10^9^ mycoplasmas/ml, with other carbohydrates (mannitol, galactose, sucrose or sorbitol; 4 g/L) was also tested to measure the effect of the sugar source on EPS synthesis. None of these sugars modified the viability of strain Afadé after 72 h of incubation (Table 1). As expected, the addition of sorbitol, which is not used as an energy source [41] or exploitable for EPS biosynthesis (due to absence of the required metabolic pathways) had no effect. Mannitol and sucrose supplements resulted in increased EPS production. This could be due to the more efficient transport and recycling of these two sugars into EPS, in comparison with glucose.

### 2. Structural Determination and Immunogenic Properties of Free EPS Secreted by *Mmm*

**Figure 3:**
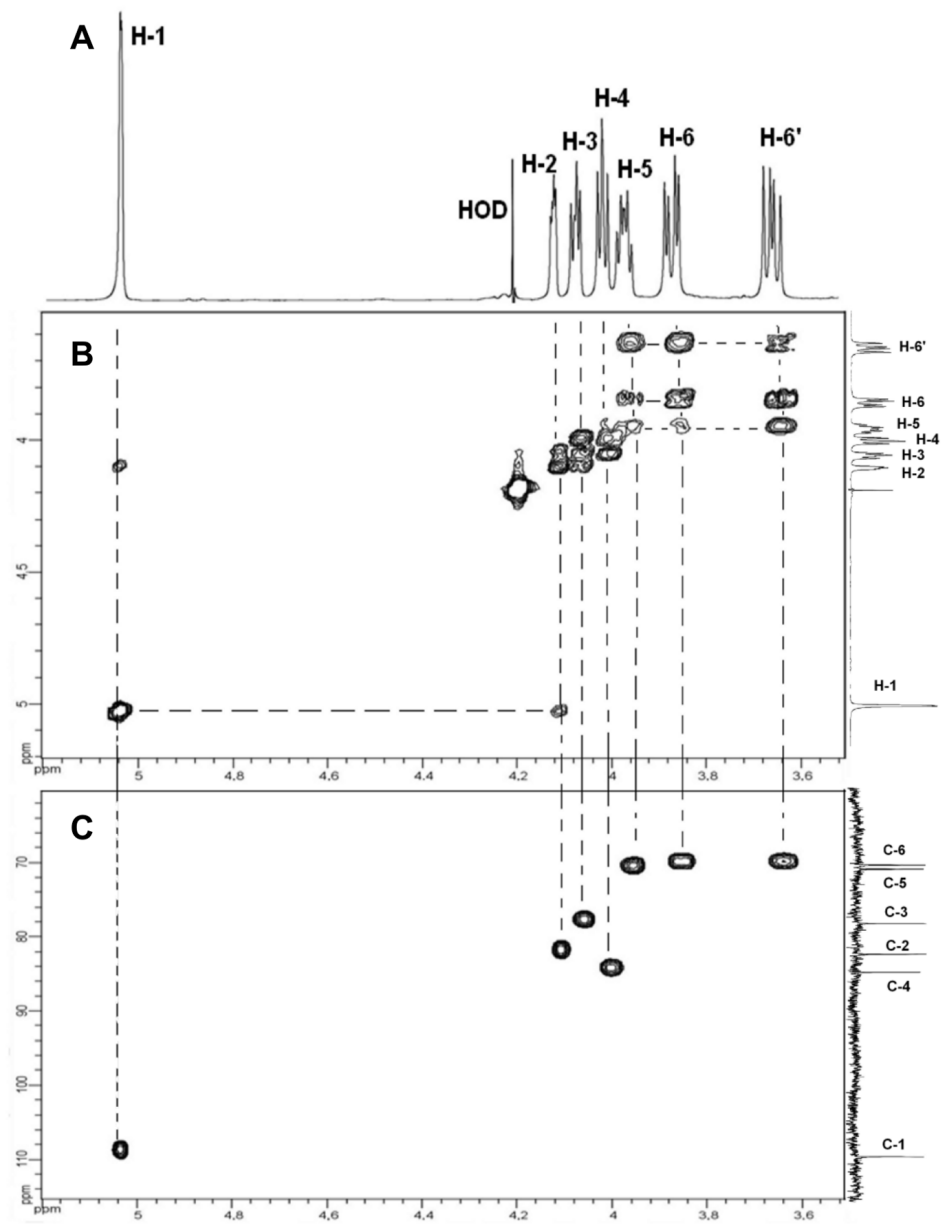
NMR spectra of EPS produced by *Mmm*. **A: ^1^H NMR spectrum, B: 2D COSY NMR spectrum and C: 2D HSQC NMR spectrum.**

*Mmm* EPS was subjected to total acid hydrolysis with 4 M trifluoroacetic acid. HPAEC-PAD analysis of the acid hydrolysate revealed the presence of galactose (96.7±1.0%) and glucose (3.3±1.0%). This composition is similar to that described by Plackett et al. for the galactan *Mmm* capsule [36]. The structural analysis of *Mmm* EPS was carried out by ^1^H and ^13^C NMR spectroscopy. The 7 resonances observed on the^ 1^H NMR spectrum (Figure 3A) and correlations detected on the 2D NMR COSY spectrum (Figure 3B) led us to attribute the observed chemical shifts to protons of galactosyl residues: 5.046 (H-1), 4.118 (H-2), 4.068 (H-3), 4.013 (H-4), 3.965 (H-5), 3.864 (H-6) and 3.648 (H-6′) ppm. On the 2D NMR HSQC spectrum (Figure 3C), the connectivities observed between H-1 and C-1 (109.0 ppm), H-2/C-2 (82.1 ppm), H-3/C-3 (78.0 ppm), H-4/C-4 (84.5 ppm), H-5/C-5 (70.8 ppm) and H-6,H-6′/C-6 (70.2 ppm) were characteristic of a galactan homopolymer. The ^13^C signal at 109.0 ppm and ^1^H signal at 5.046 ppm were typical of D-galactose residues with a β furanoside configuration [42]. Chemical shift values obtained for C-6 indicated the presence of a linkage on the C-6 in Gal residues. The ^1^H/^1^H NOESY spectrum presented interesting inter-residue connectivities: H-1 of galactose (δ 5.046) was connected to two signals at δ 3.864 and 3.648 belonging to H-6 and H-6′ of galactose. This correlation was confirmed on the ^1^H/^13^C HMBC spectrum. Thus, these connectivities and ^1^H and ^13^C chemical shifts are consistent with the literature values for a β(1−>6)-galactofuranose polymer [43]. Again, the structure of the EPS released into the culture medium was identical to that of the capsular galactan [10]. Previous studies had reported galactan to be immunogenic [12]. The EPS produced in our experimental set up was also detected by serum from a CBPP-convalescent bovine (Figure 1B). This did not represent a cross-reaction due to protein contamination of the EPS extract, since no polypeptides were detected by silver staining after SDS PAGE (data not shown).

### 3. Correlation between EPS Production and Colony Appearance

**Figure 4:**
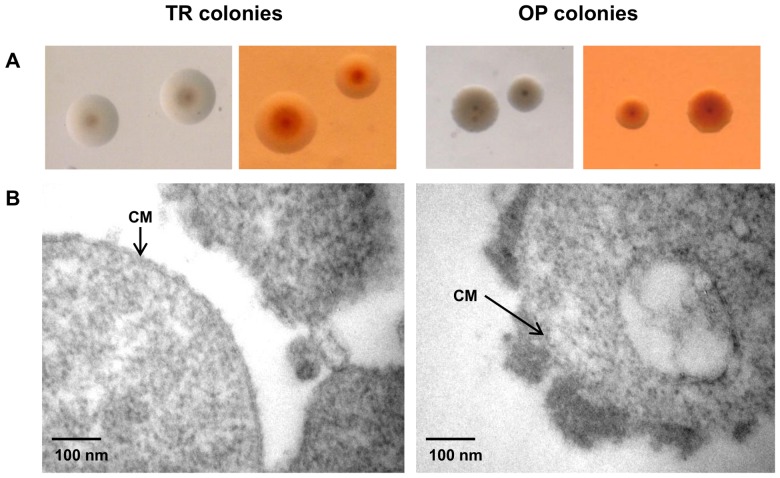
PG1^T^ colony opacity is related to the presence of capsular material. **A:** Colony morphology of PG1^T^ opaque (OP) and translucent (TR) colony variants grown on PPLO supplemented agar with (right) or without (left) Congo red. Magnification: x30. **B:** Electron microscopy of Ruthenium red-stained PG1^T^ OP and TR colonies. CM: cytoplasmic membrane.

In this study we showed that the free galactan secreted by *Mmm* strain Afadé presented the same monomeric composition and structure as the capsular polysaccharide previously described, which was associated with *Mmm* strain V5 cells [10], [36]. In other bacteria the presence of a polysaccharidic capsule has often been associated with an opaque colony phenotype [29], [44], [45]. Variation in colony opacity has also been described in mycoplasmas (*i.e. M. pulmonis* [46] and *M. hyorhinis* [47]) but not in *Mmm*. However, Minga reported the existence of rough and smooth morphotypes of *Mmm* colonies and showed that they were related both to the capacity to produce a capsule and to pathogenicity [48]. Here we showed that both *Mmm* strains PG1^T^ and Afadé produced a mixture of opaque (OP) and translucent (TR) colonies on agar (Figure 4A). TR colonies showed the typical “fried egg” appearance, with a small opaque center surrounded by a large translucent peripheral area. In contrast, OP colonies were darker and their center was not always distinct from the peripheral area. When grown on agar plates containing Congo red, a direct dye known to interact with the extracellular matrix, OP colonies showed an intense and uniform red coloration, while TR colonies were poorly stained, with staining limited mainly to their center (Figure 4A). OP and TR variants of *Mmm* PG1^T^ were then observed by electron microscopy following staining with Ruthenium red, which binds to polysaccharides. Electron micrographs obtained from TR colonies showed separated cells with scanty extracellular material, while cells from OP colonies were aggregated and coated with a patchy, electron-dense material made of polysaccharides (Figure 4B), which appeared to attach the cells together. This cellular aggregation may account for the colony opacity, as previously suggested for *Nesseria gonorrhoeae* [49].

**Figure 5:**
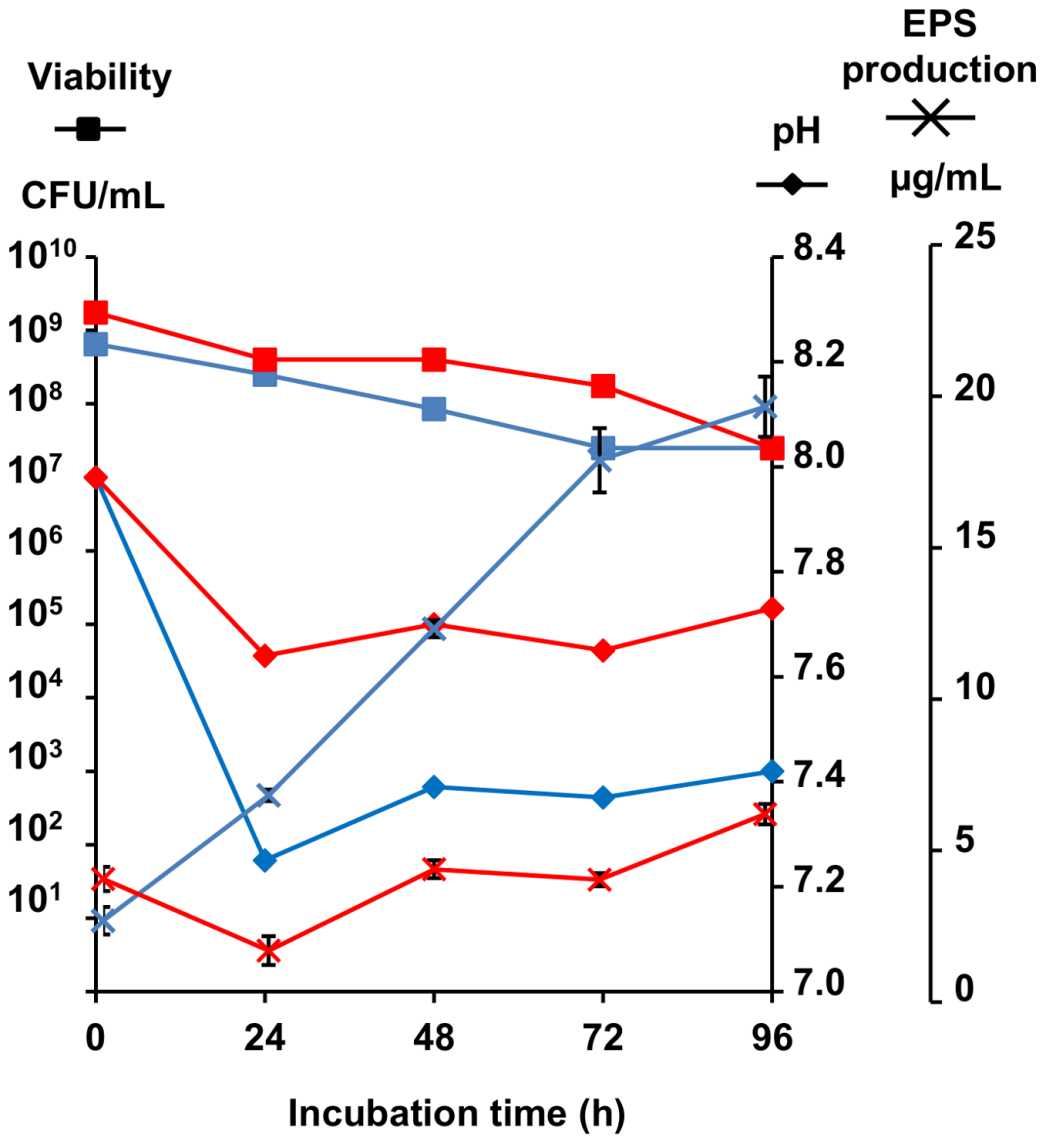
Growth, pH and exopolysaccharide (EPS) secretion of translucent (blue) and opaque (red) colony variants in CMRL-1066 medium. 50 ml of CMRL-1066 medium were inoculated with 10^8^–10^9^ CFU/mL and incubated for 96 hours. Viable titer, pH and EPS production were determined every 24 h.

**Figure 6:**
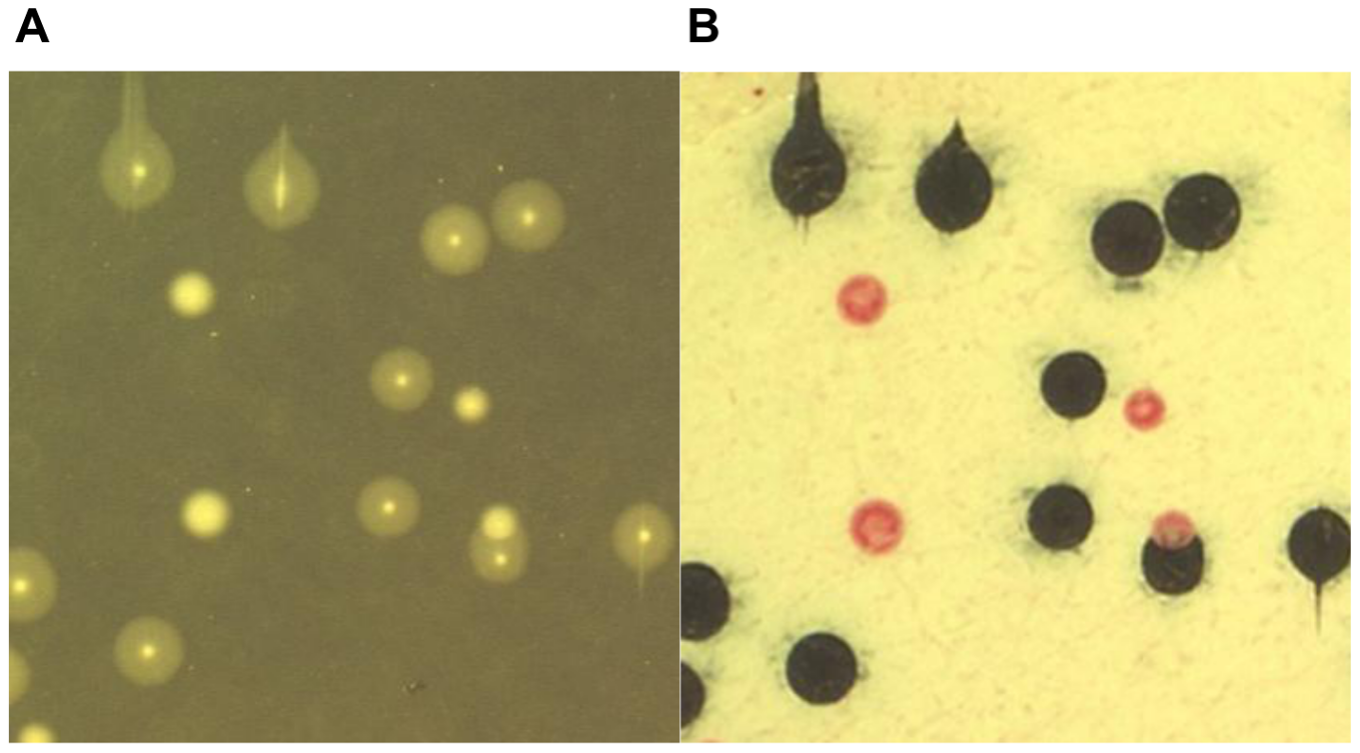
Variation in *Mmm* colony opacity associated to antigenic variation of the glucose PTS permease. **A:** *Mmm* strain Afadé colonies on PPLO supplemented agar observed under stereomicroscope with indirect light. **B:** Colony-blotting performed from the same plate using the 3F3 monoclonal antibody. (Magnification: x10).

Interestingly, OP and TR colony variants differed in their capacity to secrete EPS, with the TR yielding three-fold more EPS than the OP variant (Table 1, Figure 5). Viability in CMRL-1066 was comparable for both colony variants, though acidification of the culture medium was more pronounced for the TR variant, indicating that glycolysis was more efficient (Figure 5). Glucose metabolism is largely dependent on the capacity to import sugars and, in a previous study, we had demonstrated that a glucose permease (MSC_0860/0873), belonging to the phosphoenolpyruvate phosphotransferase system (PTS), of *Mmm* PG1^T^ was regulated by a genetic ON/OFF switch that resulted in premature termination of the PTS permease and epitope variation [23]. The monoclonal antibody targeting this epitope, namely 3F3, was shown to bind to TR but not to OP colonies (Figure 6). This may suggest that truncation of the glucose PTS permease in OP variants results in less efficient glucose transport and, consequently, less efficient glycolysis and EPS synthesis.

The frequency of reversion from 3F3-negative (3F3NEG) to 3F3-positive (3F3POS) variants of strain Afadé was shown to vary from 10^−6^ to 10^−2^ [23]. In the present study, this reversion resulted in less than 0.2% of 3F3NEG (OP colony variant) in 3F3POS cells (TR colony variant) or in *Mmm* strain Afadé cultures, and a proportion of 1 to 5% of 3F3POS (TR colony variant) cells in 3F3NEG (OP colony variant) cultures at the time of transfer into CMRL-1066 medium. This may account for the weak EPS production obtained from the OP (3F3NEG) colony variant. We then attempted to minimize the proportion of TR (3F3POS) cells in OP (3F3NEG) cultures in order to assess EPS production by the OP colony (3F3NEG) variant cells. Several cultures were tested and the one with the lowest titer of 3F3POS (TR colony variant) cells (*i.e.* 0.5%) was used to inoculate CMRL-1066 medium. Under these conditions, EPS production was very low, whatever the sugar source (Table 1). However, addition of different sugars to the CMRL-1066 medium appeared to modulate the level of reversion to 3F3POS (TR colony variant) after 72 hours of incubation. Mannitol was the only carbohydrate which led to an increase of 3F3POS (TR colony variant), from 0.5% to 12%, but the EPS release was not enhanced, due to considerable cell decay. This suggests the existence of complex regulations for transport and catabolism of the different sugars.

### 4. *In silico* Analysis of Candidate Galactan Biosynthetic Pathways

We showed that TR colony variants, which were detected by 3F3 monoclonal antibody, produced free, extracellular galactan. In contrast, OP colony variants, which did not display any reaction with the 3F3 antibody, because of a premature termination of their glucose PTS permease, produced capsular galactan. Polysaccharide biosynthesis by OP colony variants was intriguing, since truncation of the glucose PTS permease would be expected to result in an overall less efficient glucose transport and therefore hampering notably of polysaccharide synthesis.

We tried to decipher the biochemical links between glucose intake, polysaccharide chain elongation and attachment/release of the galactan by retrieving *in silico* all potentially involved *Mmm* genes. The genomes of the 2 currently sequenced *Mmm* strains, namely PG1^T^ (accession number NC_005364.2) [5] and Gladysdale (accession number CP002107.1) [6], were used.

As NMR analyses revealed that galactan is composed of galactose residues in the furanose ring form, we first looked for enzymes involved in UDP-galactofuranose synthesis, which is the usual precursor of a galactofuranose polymer. UDP-galactofuranose is usually formed from UDP-galactopyranose after the action of an UDP galactofuranose mutase Glf [50], [51]. One *glf* gene identified in *Mmm* strain PG1^T^ [5] is also present in the Gladysdale genome. In bacteria, UDP-galactopyranose can be synthesized via two enzymes of the Leloir pathways [52], either from galactose-1-phosphate after the action of a galactose-1-phosphate uridylyltransferase (GalT) or from UDP-glucose via an UDP-glucose 4-epimerase (GalE). No galT homologue was retrieved from the genomes of *Mmm*, indicating that galactose is not a source for galactan synthesis. This is concordant with our experiment showing that galactose was unable to enhance galactan synthesis (Table 1). In contrast the glucose-dependent synthetic pathway is highly probable since two genes were identified in both genomes, one of which encodes for UDP-glucose 4-epimerase (GalE) and the other for a glucose-1-phosphate uridylyltransferase (GalU) that uses glucose-1-phosphate to generate UDP-glucose.

Glucose intake in *Mmm* certainly relies on two similar (47%) glucose PTS permeases (MSC_0054 and MSC_0860/873) that have been identified in strain PG1^T^ [23] and are also present in the Gladysdale strain. The PTS glucose permeases usually allow the concomitant uptake and phosphorylation of glucose into glucose-6-phosphate [53], the typical precursor of glycolysis. The probability that glucose intake might be mediated by other generic permeases, such as ABC transporter, is low since it would require further activation of glucose by phosphorylation that is impaired because of the mutation of the two glucokinase genes in PG1^T^ [23]. Entry into the galactan biosynthetic pathway further requires the isomerization of glucose-6-phosphate to glucose-1-phosphate, which is most probably achieved by the phosphoglucomutase (ManB) predicted in *Mmm* (MSC_0829, [5]). It has been shown recently in *Vibrio cholerae* that glucose PTS permeases are general regulators of genes involved in EPS synthesis [54]. In our model, we hypothesize that MSC_0860/873 might play a role in the regulation of the attachment/release of galactan, its impairment in OP variants resulting in a constitutive attachment of the galactan.

Three predicted glycosyltransferases (GT), cps (MSC_0109), EpsG (MSC_0108) and MSC_0771 previously identified in the *Mmm* PG1^T^ genome [5] are good candidates for elongation of the galactan chain from the UDP-galactofuranose precursor. MSC_0109 (cps) showed 45% and 42% similarity with glycolipid synthases described in *M. genitalium* [55] and *M. pneumoniae* [56], respectively. In Mycoplasmas, glycolipid synthases are able to transfer galactosyl and glucosyl residues to membrane-bound diacylglycerol [57]. Therefore, MSC_0109 is a good candidate for galactan attachment to the cytoplasmic membrane. This was supported by preliminary northern blot experiments that showed a differential expression of the c*ps* gene in capsulated versus non-capsulated colony variants (Figure S1).

Functional prediction of EpsG using HHPred [34] and Phyre2 [33] revealed a structure that was highly similar (e-value = 3.4e-38, probability 100%) to that of the cellulose synthase BcsA of *Rhodobacter sphaeroides* [58]. Synthases are membrane-embedded glycosyltransferases involved in polymer formation and translocation across the cytoplasmic membrane [59]. Four potential transmembrane regions were predicted by TMHMM2 [32] for EpsG, with a large cytoplasmic loop (36–312) (Figure S2) which showed homology with family 2 glycosyltranferases (http://www.cazy.org, [31]), a group of GT known to add sugars in the β-configuration which is in agreement with the galactan structure. Two conserved motifs (DXD and QxxRW), which are common markers of processive enzymes in various bacterial models [60], were found in the cytoplasmic loop (Figure S2). Further structural analysis showed that the cytoplasmic domain was similar (e-value = 2.8e-26, probability 99.93%) to the UDP-galactofuranosyl glycotransferase GlfT of *Mycobacterium turberculosis* [61]. We therefore hypothesized that EpsG is a putative galactan synthase which could catalyze the polymerization of β-1,6-linked galactofuranose and facilitate its export across the cytoplasmic membrane. Furthermore, synthase-dependent exopolysaccharide secretion was shown to occur in the presence or absence of a lipid acceptor molecule [59]. In our model, synthase-dependent galactan synthesis might be active whether galactan is attached to a glycolipid anchor, or not.

The third predicted GT, MSC_0771, was not related to any of the 90 GT families described so far. Structural analysis revealed two domains of which only the N-terminal one (1–199) was similar to diverse bacterial and eukaryotic glycosyltransferases. Its role in galactan biosynthesis remains hypothetical.

In conclusion, this *in silico* analysis points toward i) a synthase-dependent pathway for galactan synthesis, ii) a putative role of MSC_0109 in attachment of galactan to the membrane and iii) a potential regulation of this attachment by glucose PTS permease. However detailed functional studies are now needed to unravel the precise roles of each GT and the genetic basis and triggering conditions underlying the switch between capsule production (OP colony variants) and EPS secretion (TR colony variants).

### Conclusions

We have developed a simple method for the purification of *Mmm* EPS. The incubation of washed mycoplasma cells in a completely defined medium, able to support cellular metabolism, allowed EPS synthesis for at least 72 hours, while preventing contamination with polysaccharides from the culture medium. Preliminary results in our laboratory indicate that this method is suitable for use in other mycoplasmas.

This method was applied to characterize the free EPS secreted by *Mmm*. Purified EPS had an identical β(1−>6)-galactofuranosyl structure to that of *Mmm* capsular galactan. The presence of OP and TR colony variants in *Mmm* cultures, which varied greatly in their capacity to produce free EPS, was observed. Both variants appeared to be able to synthesize polysaccharides. However, these polysaccharides either remained attached to the cells, constituting a capsule (opaque colony variants), or were released into the culture medium as free EPS (translucent colony variants). We finally conducted *in silico* analyses of *Mmm* genes potentially involved in polysaccharide biosynthesis and proposed candidate pathways that might account for the alternative production of capsular versus free galactan by the corresponding opaque and translucent colony variants.

In *Streptococcus pneumoniae* spontaneous phase variation between opaque and translucent colony variants has been associated with diverse levels of virulence at different stages of the disease, from invasion to transmission [62]. It remains to be elucidated whether variations in the production of capsular versus free galactan can regulate *Mmm* pathogenicity and how. Although free galactan has been found in the body fluids and urine of infected animals [12], [63], the occurrence and proportion of EPS versus capsule-producing variants *in vivo* has yet to be determined. This would constitute a key step in the understanding of *Mmm* pathogenicity, since the emergence of sub-populations in a given ecological niche is known to lead to bacterial persistence.

## Supporting Information

Figure S1 Northern blot hybridization of total RNA of the opaque (OP) and translucent (TR) colony variants of *Mmm* strain Afadé with a *cps* (MSC_0109) or rDNA 16S probe. Total RNA extraction and northern blot hybridization was performed as previously described [1]. The rDNA 16S probe was obtained by PCR [2]. Transcription of the rDNA 16S was used to normalize the hybridization. The *cps* gene probe was obtained by PCR with specific primers (5′ TGATGGATCAACAGATAACACCA 3′ and 5′ TTTGGGCGTGAGTATCAATAAG 3′).
(DOC)

Figure S2 Schematic representation of the predicted membrane topology of *Mmm* EpsG (MSC_0108) glycosyltransferase. Numbers indicate the localization of transmembrane helices. DxD (red) and RxxQW (blue) motifs are showed.
(TIF)
