# Supplementary material for: Characterization of Free Exopolysaccharides Secreted by Mycoplasma mycoides Subsp. mycoides
Source: PLoS One. 2013 Jul 15;8(7):e68373. doi: 10.1371/journal.pone.0068373 (PMC3711806; doi:10.1371/journal.pone.0068373)
Supplement: Figure S1 — Northern blot hybridization of total RNA of the opaque (OP) and translucent (TR) colony variants of Mmm strain Afadé with a cps (MSC_0109) or rDNA 16S probe. Total RNA extraction and northern blot hybridization was performed as previously described [1]. The rDNA 16S probe was obtained by PCR [2]. Transcription of the rDNA 16S was used to normalize the hybridization. The cps gene probe was obtained by PCR with specific primers (5′ TGATGGATCAACAGATAACACCA 3′ and 5′ TTTGGGCGTGAGTATCAATAAG 3′). (DOC) [file pone.0068373.s001.doc]

Figure S1


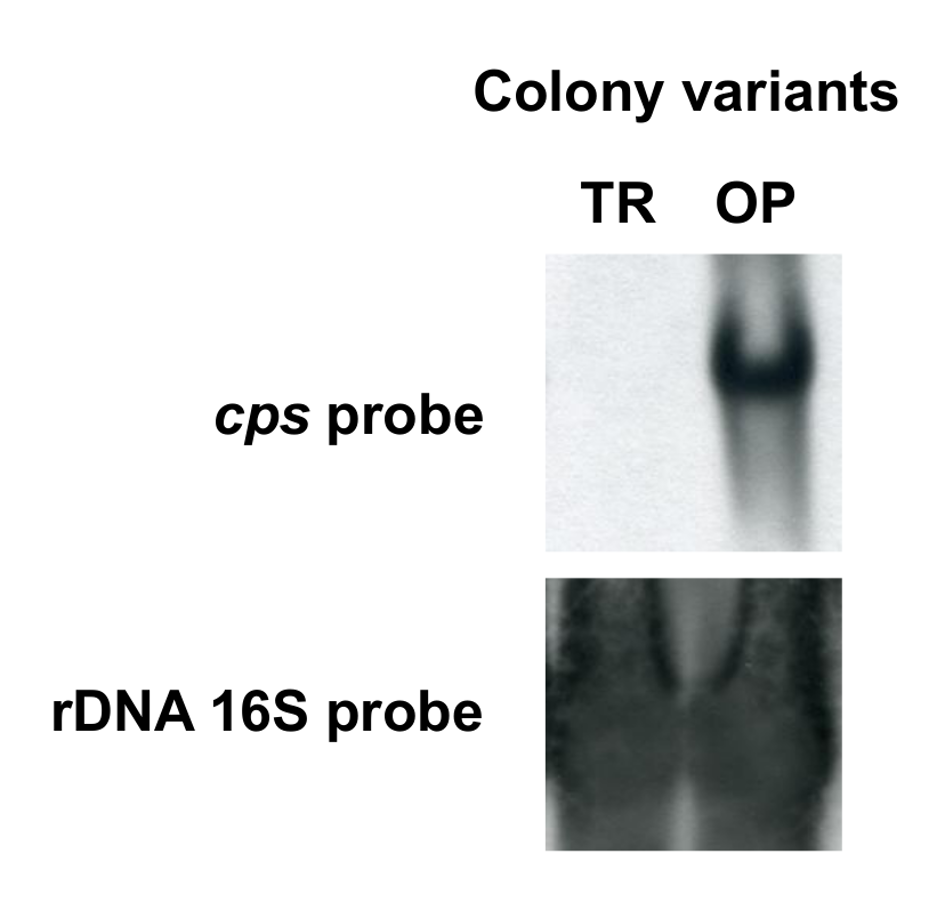


**References**

1. Gaurivaud P, Laigret F, Garnier M, Bove JM (2001) Characterization of FruR as a putative activator of the fructose operon of *Spiroplasma citri*. FEMS Microbiol Lett 198: 73-78.

2. Johansson KE, Heldtander MU, Pettersson B (1998) Characterization of mycoplasmas by PCR and sequence analysis with universal 16S rDNA primers. Methods Mol Biol 104: 145-165.
