# Supplementary figures and images for: Characterization of Free Exopolysaccharides Secreted by Mycoplasma mycoides Subsp. mycoides
Source: PLoS One. 2013 Jul 15;8(7):e68373. doi: 10.1371/journal.pone.0068373 (PMC3711806; doi:10.1371/journal.pone.0068373)

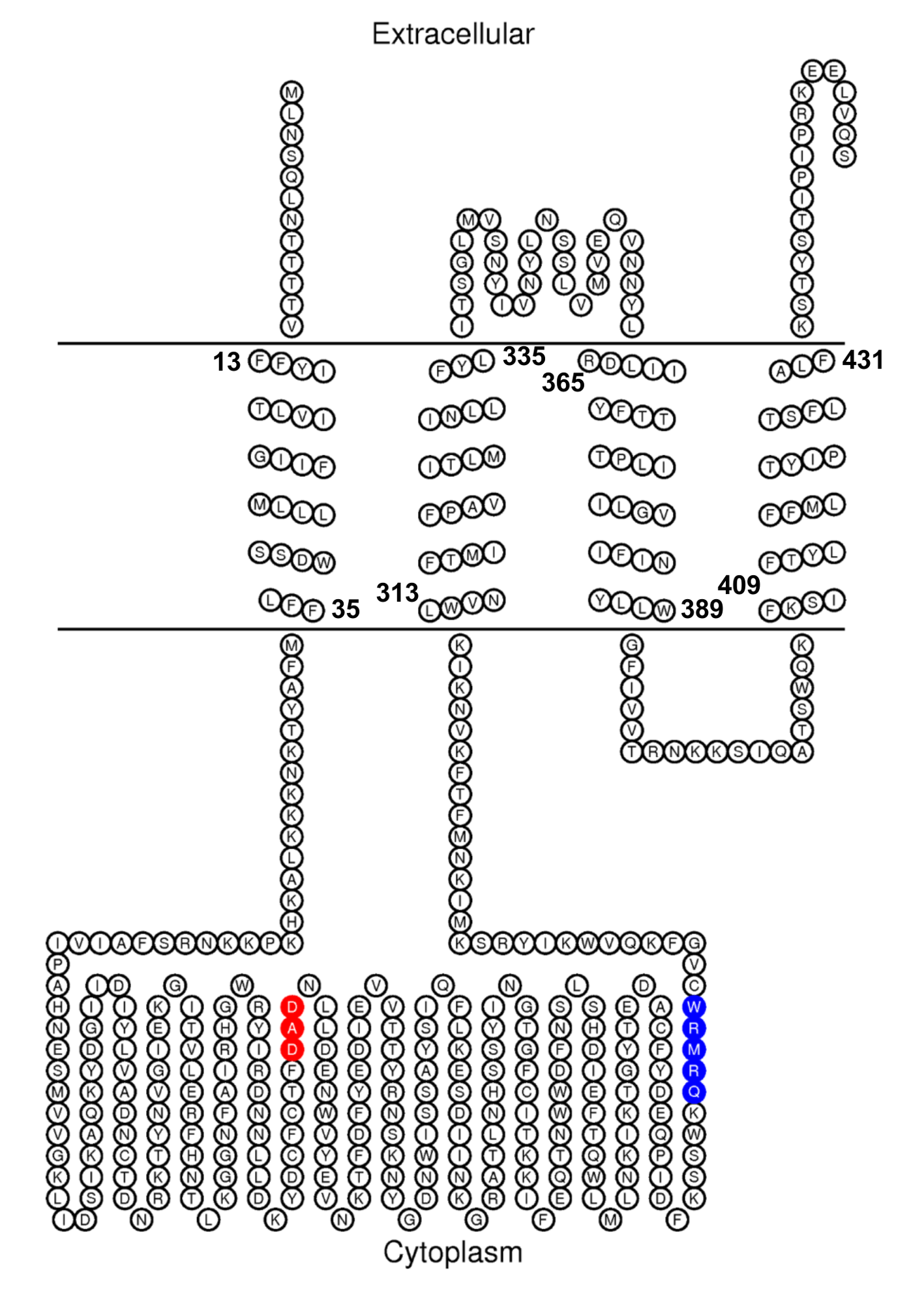

Supplement: Figure S2 — Schematic representation of the predicted membrane topology of Mmm EpsG (MSC_0108) glycosyltransferase. Numbers indicate the localization of transmembrane helices. DxD (red) and RxxQW (blue) motifs are showed. (TIF) [file pone.0068373.s002.tif]
